# The connections of Locus Coeruleus with hypothalamus: potential involvement in Alzheimer’s disease

**DOI:** 10.1007/s00702-021-02338-8

**Published:** 2021-05-03

**Authors:** Filippo Sean Giorgi, Alessandro Galgani, Stefano Puglisi-Allegra, Carla Letizia Busceti, Francesco Fornai

**Affiliations:** 1 Department of Translational Research and New Technologies in Medicine and Surgery, University of Pisa, Via Roma 55, 56126 Pisa, Italy; 2 Neurology Unit, Pisa University Hospital, 56126 Pisa, Italy; 3 I.R.C.C.S. Neuromed, Via Atinense 18, 86077 Pozzilli, IS Italy

**Keywords:** Locus Coeruleus, Hypothalamus, Noradrenaline, Sleep, Autonomic system, Neurodegenerative disorders, Alzheimer’s disease

## Abstract

The hypothalamus and Locus Coeruleus (LC) share a variety of functions, as both of them take part in the regulation of the sleep/wake cycle and in the modulation of autonomic and homeostatic activities. Such a functional interplay takes place due to the dense and complex anatomical connections linking the two brain structures. In Alzheimer’s disease (AD), the occurrence of endocrine, autonomic and sleep disturbances have been associated with the disruption of the hypothalamic network; at the same time, in this disease, the occurrence of LC degeneration is receiving growing attention for the potential roles it may have both from a pathophysiological and pathogenetic point of view. In this review, we summarize the current knowledge on the anatomical and functional connections between the LC and hypothalamus, to better understand whether the impairment of the former may be responsible for the pathological involvement of the latter, and whether the disruption of their interplay may concur to the pathophysiology of AD. Although only a few papers specifically explored this topic, intriguingly, some pre-clinical and post-mortem human studies showed that aberrant protein spreading and neuroinflammation may cause hypothalamus degeneration and that these pathological features may be linked to LC impairment. Moreover, experimental studies in rodents showed that LC plays a relevant role in modulating the hypothalamic sleep/wake cycle regulation or neuroendocrine and systemic hormones; in line with this, the degeneration of LC itself may partly explain the occurrence of hypothalamic-related symptoms in AD.

## Introduction

Alzheimer’s disease (AD) is the most frequent cause of cognitive impairment worldwide, with memory deterioration representing its clinical core (Dubois et al. 2014). Nonetheless, in AD patients also other symptoms are commonly observed, such as sleep disorders, autonomic disfunction, and endocrine alterations (Ishii and Iadecola 2015). These non-cognitive symptoms may be partly explained in the light of the involvement of the hypothalamus in the neurodegenerative process occurring in AD.

The hypothalamus is considered as the master regulator of homeostatic and vegetative functions; it controls the neuroendocrine axis, modulates sympathetic and parasympathetic activities and takes part in the complex neuronal networks involved in the sleep/waking cycle (Lechan and Toni 2000). Several pathological pieces of evidence support the hypothesis that the hypothalamus might be involved early and significantly in AD. Among them, are particularly worth mentioning the observations by Braak and colleagues in their extensive studies on tau-related pathology in AD (Braak et al. 2011), in which they clearly showed that the hypothalamus is one of the brain regions mostly and earlier affected by degenerative phenomena; indeed, they observed that abnormal accumulation of hyperphosphorylated tau protein (pTau) occurs in the hypothalamic tuberomammillary nucleus (TMN) already at the so-called “stage Ic” of Braak pTau AD staging, i.e. in the earliest phases of tau-related AD pathology development, decades before cortical involvement (Braak et al. 2011).

The noradrenergic nucleus Locus Coeruleus (LC) is the main noradrenergic (NA) nucleus of the brain, providing NA innervation for the whole cortical mantle and many subcortical structures (Poe et al. 2020). LC-NA system significantly contributes to several brain functions; it promotes brain homeostasis, modulating neurovascular unit functioning and neuronal-glial interaction, it takes part in a variety of cognitive processes, ranging from focused attention to learning and memory formation, and it plays a crucial role in modulating the sleep-waking cycle (Aston-Jones and Cohen 2005; Lecrux and Hamel 2016; Foote and Berridge 2019; Giorgi et al. 2020a). LC has been receiving growing attention in the last years for its possible pathogenic and pathophysiological role in AD (Giorgi et al. 2017; Kelly et al. 2017), since it may be markedly involved since the earliest stages of the disease (Braak et al. 2011). Detailed descriptions of both LC and hypothalamus functional anatomy are beyond the specific aims of this review, and these topics are described elsewhere (for specific reviews, see, for instance, Counts and Mufson 2012; Saper 2012).

LC sends specific projections to the hypothalamus, and through these it modulates a variety of hypothalamic functions. In line with this, the disruption of LC-hypothalamic connections might explain, at least in part, some of the non-cognitive symptoms occurring in AD. Furthermore, some hints coming from experimental studies and from post-mortem human studies, suggest that LC-NA system degeneration may also promote degenerative processes in several brain regions, including the hypothalamus. In this review, we will first analyze the available literature on LC-hypothalamic involvement in AD and discuss its possible role in its pathogenesis, then we will summarize the anatomical and functional connections which link the LC with the hypothalamus and how their disruption may contribute to AD pathophysiology.

## LC degeneration in AD

LC involvement in AD has been shown decades ago (Mann et al. 1980; Kelly et al. 2017). The first studies date back to the 1980s of the last century when different authors found that a dramatic degeneration of LC and its fibers could be observed in brain specimens from AD demented patients (Mann et al. 1980, 1982, 1984; Scheibel et al. 1987; German et al. 1992). However, those studies were performed in patients suffering from advanced AD stages and did not profit from accurate histological analysis. More recent studies provided further pieces of evidence confirming and strengthening the former observations; in particular, in 2011, Braak’s group released the revised classification of neurofibrillary tangles (NFT)-pathology in AD, based on the evaluation of a huge number of AD brains and stated that LC is the first brain structure to show tau-related pathology, decades before neocortical involvement (Braak et al. 2011). In 2013, Wilson and colleagues showed that LC integrity was strongly associated with cognitive performances, from the milder stages of cognitive impairment (Wilson et al. 2013); in 2017, Theofilas and colleagues found that LC is not affected by degenerative phenomena during physiological ageing, contrarily to what had been previously suggested by others (Manaye et al. 1995), but that rather its damage is strictly associated with AD pathology (Theofilas et al. 2017). In the same year, Kelly et al. performed a pivotal study by profiting of an accurate stereological analysis to estimate the true number of neurons within LC in subjects which had been closely followed-up neurologically during their life (Kelly et al. 2017). They observed that a marked and significant damage of LC can be observed in AD patients already at the mild cognitive impairment (MCI) stage; moreover, the degree of LC degeneration was found to be strictly correlated with the cognitive performances of the subjects (Kelly et al. 2017). Therefore, the current knowledge on LC involvement in AD seems to point toward a crucial role of this nucleus, which may be particularly relevant in its pathogenesis from the earliest stages of AD.

## The occurrence of neuronal loss/degenerative phenomena and NA alterations in the hypothalamus of AD patients

Hypothalamus degeneration is a well-established feature of AD (Swaab et al. 1992; Thal et al. 2002; Braak et al. 2011). Several post-mortem studies have shown the occurrence of neuronal loss together with Aβ_42_ plaques and NFT deposition in several hypothalamic nuclei, in particular the suprachiamastic nucleus (SCN), the supraoptic nucleus (SON), the paraventricular nucleus (PVN) and the TMN (Mann et al. 1985; Braak and Braak 1991; Baloyannis et al. 2015). In line with this, in vivo magnetic resonance imaging (MRI) studies showed reduced hypothalamic volume and gray matter loss in patients suffering from AD when compared to healthy controls (Callen et al. 2001; Whitwell et al. 2007). In the first part of each one of four sub-sections which follow section “Connections between the LC and the hypothalamus: potential functional effects of LC loss”, a detailed description of the involvement of specific hypothalamic nuclei in AD will be provided.

Histological post-mortem studies performed in the 1980s of the last century showed that NA innervation of the hypothalamus was reduced in AD (Mann et al. 1980; Aral et al. 1984). As already said, LC is the main source of hypothalamic NA innervation (Farley and Hornykiewicz 1977; Bowden et al. 1978); thus, it is likely that NA hypothalamic loss observed in AD is mainly due to the degeneration of LC itself. In particular, in 1985 Mann and colleagues assessed the occurrence of degenerative phenomena both in LC and hypothalamus of AD patients; they found that the damage of LC was associated with atrophy and neuronal loss in the SON and PVN, and discussed these alterations as related to the loss of NA projections from the LC to the hypothalamus (Mann et al. 1985).

In the section that follows, and in its two sub-sections, based on available literature, we will discuss how the LC impairment may concur to hypothalamus degeneration; in fact, these two pathological events (i.e. LC and hypothalamus degeneration) may not be merely concomitant, but indeed connected, at least in part, by a causative link.

## The role of LC degeneration in the pathogenesis of neurodegeneration occurring in AD

LC seems to be particularly prone to neurodegeneration and such a susceptibility may be explained by several aspects, including the oxidative stress caused by NA metabolism, in parallel with the metabolic vulnerability due to the high neuronal activity of LC along the lifespan (for an extensive review, see Weinshenker 2018). In line with LC degeneration, NA levels are significant reduced in LC target areas, including the amygdala and hippocampus, among the limbic structures, as well as in the hypothalamus among subcortical structures (Farley and Hornykiewicz 1977; Aral et al. 1984). The administration of the neurotoxin DSP-4, a neurotoxin which is highly selective for LC axon terminals when injected systemically (Fritschy and Grzanna 1991), reproduces a similar pattern of NA loss in rats (Table 2) and for this reason this experimental approach can be helpful in disclosing at least some of the phenomena which are due to LC loss in patients affected by neurodegenerative diseases (NDDs) (see below).

The consequences of LC-NA system impairment on its target regions should be considered under both a functional and a pathogenetic point of view. Regarding the former aspect, NA is a ubiquitous neurotransmitter and is key in modulating several brain functions from the cellular up to the neural network level (Poe et al. 2020). Thus, the loss of LC-NA is likely to be significantly involved in a variety of neurological symptoms occurring in NDDs, such as memory impairment (Hou et al. 2019), behavioral and mood disorders (McCall et al. 2017; Seki et al. 2018), and sleep alterations (Berridge et al. 2012). However, a growing amount of literature supports the hypothesis that LC degeneration may play also a pivotal pathogenetic role in AD (Weinshenker 2018), which might be due to both the dysregulation of brain homeostasis (Giorgi et al. 2020a, b), and to a direct contribution to worsening of the underlying proteinopathy and neuronal cell loss which characterizes these brain disorders (Heneka et al. 2006; Iba et al. 2015).

### LC and tau pathology spreading: the potential involvement of hypothalamus

LC is considered as one of the main key points in the hierarchical and progressive involvement of brain structures occurring in NDDs, a hypothesis which suggests that neurodegenerative proteins, such as tau or amyloid, may even spread throughout neuronal pathways in a prion-like fashion (Braak and Del Tredici 2011). Thus, the widespread projection system of LC may represent an ideal pathway through which pathological proteins could potentially reach several brain structures, as shown in an experimental model of tauopathy by Iba and colleagues (Iba et al. 2015). In line with this, a variety of neuropathological classifications of tauopathies and synucleinopathies include the LC as a brain structure involved already in the first stages of degenerative phenomena (Braak et al. 2003, 2011; Kovacs et al. 2020). It is worth noting that, regarding tauopathies, a very interesting piece of data was obtained by Kang and colleagues in 2020, which showed that 3,4-dihydroxyphenylglycolaldehyde, a metabolite deriving from the degradation of NA by monoaminoxidase-A, is able to promote tau cleavage into the specific isoform which is more prone to aggregation and propagation (Kang et al. 2020). In this same study performed in mice, these authors also showed that pathological tau tended to spread to the whole brain along LC projections (Kang et al. 2020).

Such a finding might be of particular interest, especially when considering the revised Braak’s classification of tau pathology in AD (Braak et al. 2011). According to Braak and colleagues, the AD-related tau pathology may start in the LC, decades before the clinical onset of the disease, and from this nucleus, it may spread toward the entorhinal cortex and hippocampus and, then, to the whole neocortex (Braak et al. 2011). In line with this, it is very interesting to note that in the same Braak’s classification, the authors reported that the TMN is one of first structure to be involved by tau pathology, after LC; indeed, pTau accumulation within TMN neurons can be observed already at the stage “Ic” of Braak pTau pathology staging, i.e. before cortical involvement, in the third-fourth decade of life (Braak et al. 2011). Thus, it may be hypothesized that the spreading of pTau through LC projections leads to the occurrence of tau-related pathology into the hypothalamus. The already cited study by Iba and colleagues further strengthens this hypothesis (Iba et al. 2015); in this study, the authors injected synthetic tau fibrils into the LC of PS19 tau transgenic mice, a well-known animal model of tau pathology. They observed that a few weeks after the injections, pTau starts to accumulate within several brain structures, including the hypothalamus; moreover, such an accumulation was found to progressively increase with time, in association with the worsening of tau-related pathology (Iba et al. 2015).

As a proof of concept of such a hypothesis, human post-mortem studies had shown that the hypothalamus suffers from a relevant burden of tau-pathology in AD, especially considering the nuclei involved in the regulation of circadian rhythm and the sleep/wake cycle (see “Circadian rhythm and sleep/wake cycle: potential role of LC-hypothalamic interactions” section), such as the already mentioned TMN, but also the SCN, the dorsomedial nucleus (DMN) and the ventromedial nucleus (VMN) (Hiller and Ishii 2018).

### The loss of neuroprotective effects of LC efferences may enhance AD pathology also in the hypothalamus

The impairment of LC may bear other detrimental effects in AD pathogenesis, besides its role in tau pathology spreading. In fact, LC-NA system plays an important neuroprotective role in physiological conditions, regulating microglia activity and modulating neurovascular unit functioning (Giorgi et al. 2020a; Heneka et al. 2015).

In particular, LC-NA plays an anti-inflammatory effect, and its lesion increases neuroinflammation (Giorgi et al. 2020b). The experimental lesion of LC by DSP-4 in mice models causes an increase in pro-inflammatory cytokines secretion (Feinstein et al. 2002; Heneka et al. 2003) and aberrant activation of microglial cells (Heneka et al. 2010; Jardanhazi-Kurutz et al. 2011), which is associated with a reduced efficacy in amyloid phagocytosis and clearance in AD models (Heneka et al. 2006). Moreover, LC exerts an important role in the function of neurovascular unit, both by regulating cerebral blood flow and by modulating the activity of the blood–brain barrier (BBB) (Giorgi et al. 2020a). LC lesion induces the disruption of BBB and cerebral blood flow imbalance, thus causing a condition of relative hypoxia; both phenomena contribute to neurodegenerative processes, by promoting neuroinflammation, worsening cellular damage and abnormal protein accumulation (Kalinin et al. 2006; Zlokovic 2011; Bekar et al. 2012; de la Torre 2017; Giorgi et al. 2020a; Yu et al. 2020). In line with this, intriguingly, in AD transgenic mice LC lesion has been associated with increased cerebral amyloid angiopathy and capillary pathology (Kelly et al. 2019). Furthermore, as already mentioned, the impairment of microglial phagocytosis and the BBB breakdown may result into the failure of the abnormal protein clearance; such an inference has been confirmed in animal models of cerebral amyloidosis or tauopathy, in which the experimental lesion of LC induces a marked increase of pathological burden and abnormal protein accumulation (Heneka et al. 2006; Chalermpalanupap et al. 2017).

All these mechanisms may promote hypothalamus degeneration in AD. It is worth to note that two different studies found that hypothalamic neurons may be particularly sensitive to neuroinflammation (Grossberg et al. 2011; Zhang et al. 2013). In 2011, Grossberg and colleagues observed that in mice exposed to endotoxin, the activation of inflammatory system caused the inhibition of orexinergic cells activity; in 2013, Zhang and colleagues obtained similar results concerning the gonadotropin releasing hormones (GnRH) producing cells of the medial preoptic nucleus (MPO) (Grossberg et al. 2011; Zhang et al. 2013). On the other hand, while no specific data are available regarding AD-related vascular pathology in hypothalamus, it is well-known that this brain structure is markedly affected by cerebral amyloidosis (Swaab et al. 1992; Thal et al. 2002; Hiller and Ishii 2018). According to Thal’s staging of amyloid deposition, hypothalamus is involved from the “stage 3”, i.e. before brainstem structures and right after the neocortex and the thalamus (Thal et al. 2002); in particular, amyloid plaques seems to accumulate within the SCN, the PVN and the TMN (Swaab et al. 1992; Hiller and Ishii 2018), though all the hypothalamic nuclei show amyloid degeneration (Thal et al. 2002). Thus, it is possible that LC degeneration may hamper hypothalamic functioning also by this mechanism, i.e. by primarily triggering neuroinflammation and exacerbating amyloid accumulation.

Finally, LC-NA strongly modulates the production of growth factors in its target regions, which are key in protecting from neurodegeneration (Follesa and Mocchetti 1993). Even though there are no specific studies assessing the connection between LC and hypothalamic growth factor levels, there are evidences for their decrease in AD (Claudio Cuello et al. 2019) which might be in line with such a hypothesis; thus, this might be a further mechanism explaining the link between LC loss and hypothalamic degeneration.

## Connections between the LC and the hypothalamus: potential functional effects of LC loss

**Fig. 1:**
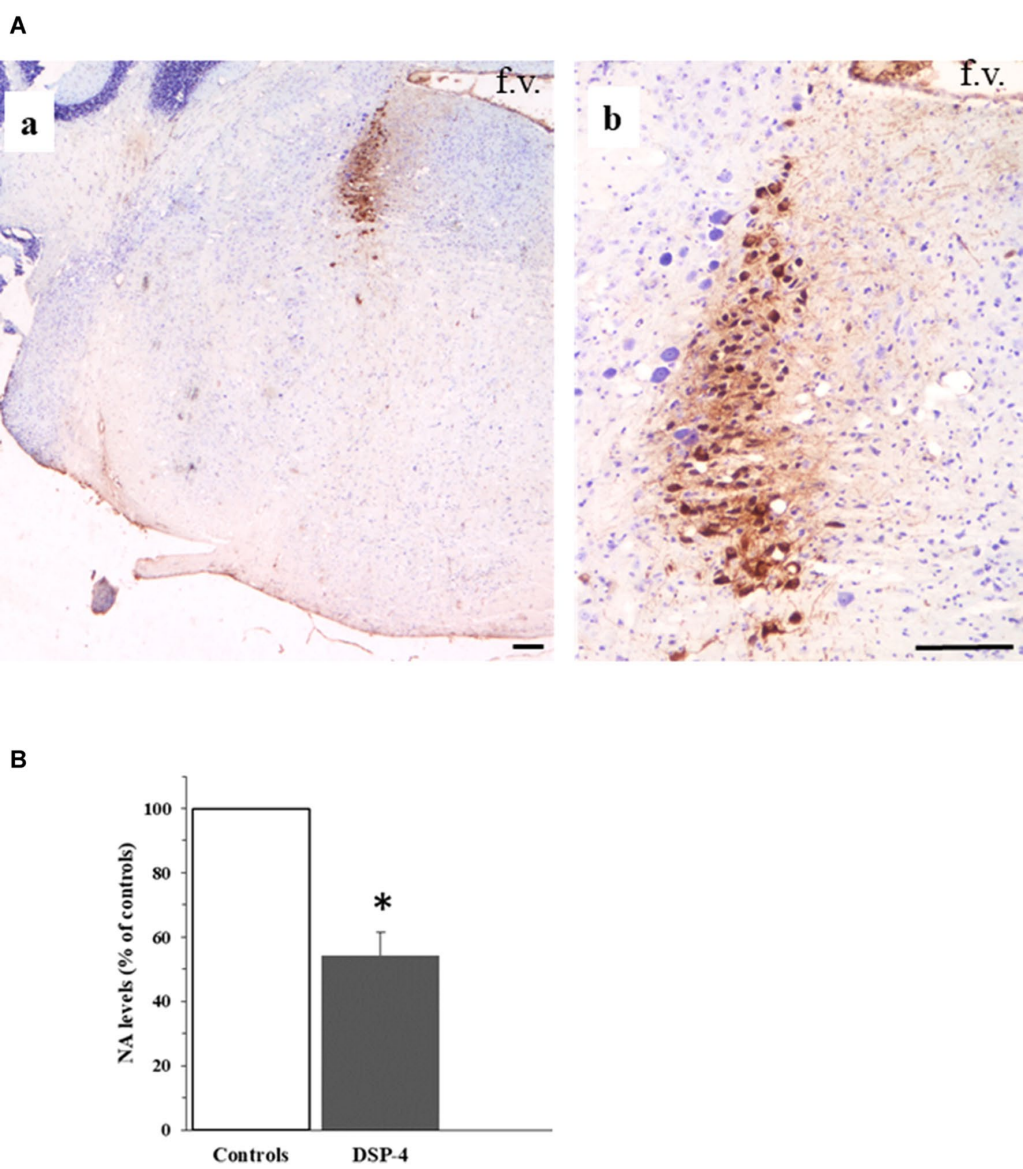
The Locus Coeruleus. The figure in panel **a** shows two pictures at different magnifications (**a** and **b**) of a 10 µm-thick paraffin-embedded coronal section cut at the level of the pons from the brain of an adult C57 Black male mouse (Charles River). The section is collected at approximately at − 5.3 mm from the Bregma, according to the stereotactic mouse brain atlas by Paxinos and Franklin (2001). The section has been immune-stained with a primary antibody (#T1299 Sigma, U.S.A.) against the enzyme tyrosine hydroxylase (TH). Neurons immune-positive for the enzyme TH (brown color in the figure, due to DAB staining of biotin-coupled anti-mouse antibodies followed by exposure to Horseradish peroxidase streptavidin; Vector Laboratories), are neurons belonging to the nucleus Locus Coeruleus (LC); the section is counter-stained with Nissl Staining (Cresyl violet). The LC nucleus is placed right below the floor of the fourth ventricle of the pons (abbreviated as “f.v.” in the pictures) (scale bar: 200 µm). The graph in panel **b** shows the effects of the experimental lesion of LC-hypothalamic projections by the neurotoxin *N*-(2-chloroethyl)-*N*-ethyl-2-bromobenzylamin (DSP-4). The systemic administration of DSP-4 selectively lesions NA terminals originating from the LC in rodents. The figure shows the effect of the administration of DSP-4 50 mg/kg i.p. in adult Sprague Dawley Rats (DSP-4 *N* = 5; controls *N* = 5) on NA levels in homogenates collected from the hypothalamus (see legend to Table 2 for details on methodology). The NA levels (ng/mg protein) of the group “DSP-4” are expressed as % of “controls”. *p < 0.01 vs controls

As said, LC and hypothalamus are densely interconnected with each other. The efferent fibers originating from the LC reach forebrain structures through two bundles, namely the ventral and the dorsal noradrenergic ones (Szabadi 2013). These neural pathways travel along the mesencephalon, reaching the forebrain and widely distributing to its structures; according to lesioning studies, the majority of NA fibers reach the hypothalamus through the dorsal noradrenergic bundle (Samuels and Szabadi 2008). Table 1 reports a detailed list of the anatomical connections between LC and hypothalamus which have been documented by experimental studies in animal models as well as by human post-mortem studies. It is worth noting that, in rodents, the experimental selective lesion of LC by DSP-4 induces a significant decrease of NA levels at the level of the hypothalamus (Fig. 1; Table 2).

**Table 1:** Anatomical connections between Locus Coeruleus and hypothalamus which have been documented by experimental/post-mortem studies

Study	Animal	Method	Hypothalamus				Preoptic		Supraoptic						Median			Mammillary	
			Ant	Post	Med	Lat	MPO	LPO	PeVN	SCN	PVN	AN	LHN	SON	DMN	VMN	ArN	PN	TMN
Mizuno, 1970	Rabbits	Electrolytic lesion of hypothalamus and EM observation of cellular debris	AFF	AFF	AFF	AFF													
Mizuno, 1972	Rabbits	Electrolytic lesion of hypothalamus and EM observation of cellular debris			AFF	AFF													
Ross, 1974	Rats	Electrolytic lesion of LC and DBH staining				EFF													
Kobayashi, 1974	Rats	Electrolytic lesion of LC and NA concentration assay							EFF		EFF								
Roizen, 1976	Rats	Stereotaxic lesion of dorsal NA bundle and NA concentration assay							EFF										
McBride, 1976	Cats	Electrolytic lesion of LC and FH staining				EFF								EFF					
		6-OHDA lesion of LC and FH staining				EFF													
		Injection of radiolabeled proline in the LC				EFF													
		HRP (retrograde tracing)				EFF										EFF			
Worth, 1976	Rats	6-OHDA lesion of LC and NA concentration assay	EFF	EFF	EFF	EFF													
Swanson, 1976	Rats	Injection of radiolabeled proline in the MPO					AFF												
Saper, 1976	Rats	Injection of radiolabeled proline in the VMN														AFF			
Farley, 1977	Humans	NA concentration assay in post-mortem brain sample	EFF	EFF		EFF								EFF					EFF
Jones, 1977	Rats	NA concentration assay; injection of radiolabeled proline	EFF	EFF	EFF	EFF													
Levin, 1977	Rats	Injection of 3H-fucosyl-glycoprotein in the LC	EFF	EFF															
Jones, 1977	Rats	Injection of radiolabeled amino acid				EFF			EFF		EFF			EFF					
Cedarbaum, 1978	Rats	HRP into hypothalamus				AFF					AFF		AFF		AFF	AFF		AFF	
Levin, 1978	Rats	Injection of radiolabeled leucine in the LC	EFF	EFF															
Bowden, 1978	Rhesus monkeys	Injection of radiolabeled proline in the LC				EFF					EFF			EFF	EFF				
Cimarusti, 1979	Rats	DBH staining of hypothalamus							EFF		EFF		EFF						
Saper, 1979	Rats	Injection of radiolabeled proline in the lateral hypothalamic nucleus											AFF						
Clavier, 1979	Rats	Injection of HRP in the LC											AFF			AFF			
Mason, 1979	Rats	Injection of HRP in the hypothalamus	EFF	EFF	EFF	EFF													
Iijima, 1980	Rats	Injection of HRP in the SON												EFF					
Palkovits, 1980	Rats	Lesion of NA nuclei (not only LC) and NA concentration assay							EFF		EFF			EFF			EFF		
		Anterograde tracing with CBT or PHLA in hypothalamus					AFF	AFF					AFF		AFF				
Barone, 1981	Rats	Injection of HRP in the lateral hypothalamus											EFF						
McKellar, 1981	Rats	Injection of radiolabeled amino acid in the LC									EFF								
Sawchenko, 1981	Rats	Both injection of radiolabeled amino acid in the LC and retrograde tracing with TB									EFF			EFF					
Kita, 1982	Rats	Injection of HRP in the lateral hypothalamus				EFF													
Veazey, 1982	Monkeys	Injection of radiolabeled amino acid in the posterior hypothalamus																AFF	AFF
Hawthorn, 1985	Rats	Electric stereotaxic lesion of paraventricular, supraoptic and suprachiasmatic nuclei and VPA concentration assessment								AFF	AFF			AFF					
Tribollet, 1985	Rats	Injection of HRP in the supraoptic nucleus												EFF					
		Injection of TB in the supraoptic nucleus												EFF					
Logue, 1985	Rats	Intraperitoneal DSP4 administration and NA concentration assessment	EFF	EFF	EFF	EFF													
Jones, 1985	Rats	Injection of radiolabeled leucine in the LC	EFF	EFF	EFF	EFF													
Javitch, 1985	Rats	Mazindol autoradiography via desipramine inhibition of NET							EFF		EFF			EFF			EFF		
Sawchenko, 1985	Rats	Retrograde tracing with TB and immunoreactivity for DBH and NPY									EFF								
Alonso, 1986	Rats	Injection of radiolabeled leucine in the SON												AFF					
Loughlin, 1986	Rats	Injection of HRP in the hypothalamus	EFF	EFF	EFF	EFF													
Loughlin, 1986 (b)	Rats	Injection of HRP in the hypothalamus	EFF	EFF	EFF	EFF													
Shirokawa, 1987	Rats	Electrode stimulation of LC and antidromic assessment of DMN													AFF				
Rizvi, 1994	Rats	Anterograde tracing with PHLA/WGA-HRP					AFF												
		Retrograde tracing with FG/WGA-HRP					AFF												
Canteras, 1994	Rats	Injection of HRP in the DMN													AFF	AFF			
Zardetto-Smith, 1995	Rats	Injection of PHAL in MPO and DBH staining					AFF												
Luppi, 1995	Rats	Injection of CTB in the LC					AFF	AFF		AFF	AFF		AFF		AFF		AFF		
Peyron, 1998	Rats	Immunohistochemical assay of ORX fibers											AFF (ORX)		AFF (ORX)			AFF (ORX)	AFF (ORX)
Horvath, 1999	Rats	Immunohistochemical assay of ORX fibers											AFF (ORX)		AFF (ORX)			AFF (ORX)	AFF (ORX)
	Monkeys	Immunohistochemical assay of ORX fibers											AFF (ORX)		AFF (ORX)				
Steininger, 2001	Rats	Injection of BD into the ventral hypothalamus					AFF	AFF											
Aston-Jones, 2001	Rats	Transfection with Pseudorabies virus of LC								AFF					AFF				
Krout, 2002	Rats	Injection of CTB and Pseudorabies virus in the SCN								AFF									
Chou, 2002	Rats	Injection of CTB in the preoptic area of hypothalamus						EFF											
		Injection of BD, PHAL, WGA-HRP in the preoptic area						AFF											
Baldo, 2003	Rats	Immunohistochemical assay for DBH									EFF				EFF	EFF			
		Injection of BD and PHAL into the LC									EFF								
Deurveilher, 2005	Rats	Injection of CTB and BD into the MPO, DMN and PVN					AFF								AFF				
Lee, 2005	Rats	Injection of FG and WGA into the LC					AFF	AFF					AFF				AFF	AFF	
Espana, 2005	Rats	Injection of WGA into the LC											AFF (ORX)						
Reyes, 2005	Rats	Injection of BD and PHAL into the PVN									AFF								
Campbell, 2007	Rats	Transfection of viral vector into the GnRH releasing neurons of preoptic area of hypothalamus					EFF												
Uschakov, 2007	Rats	Injection of BD into the MPO					AFF												
		Injection of FG into the LC					AFF												
Puskas, 2010	Rats	Immunohistochemical assay of ORX fibers											AFF (ORX)		AFF (ORX)				
Geerling, 2010	Rats	Injection of PHAL into the PVN									AFF								
Sobrinho, 2011	Rats	Injection of FG into the median/mammillary area of hypothalamus													EFF			EFF	EFF
Dimitrov, 2013	Mice	Injection of FG into the LC									AFF	AFF	AFF (GABA)			AFF		AFF (GABA)	
		Injection of BD into hypothalamus									AFF	AFF	AFF (GABA)					AFF (GABA)	
Yoon, 2013	Rats	Injection of green RetroBeads™ into the LC															AFF (NPY)		

The table reports all the available studies performed in animals or in humans assessing the anatomical connections between Locus Coeruleus and the hypothalamus; first author, year of publication, animal model and tracing technique are reported in the first three columns on the left. In the following columns, observed connections are enlisted under the generic label of “hypothalamus” or under the specific labels of hypothalamic area/nucleus explored, according to reported results. When specified in the study, the neurotransmitter released by hypothalamic projections is reported

*AFF* afferences from hypothalamus or hypothalamic nuclei to the LC, *Ant* anterior hypothalamus, *AN* anterior nucleus, *ArN* arcuate nucleus, *BD* biotinylated dextran, *CTB* cholera toxin B, *DBH* dopamine beta-hydroxylase, *DMN* dorsomedial nucleus, *DSP-4* *N*-(2-chloroethyl)-*N*-ethyl-2-bromobenzylamine, *EFF* efferences from the LC to hypothalamus or hypothalamic nuclei, *EM* electronic microscopy, *FH* fink-heimer, *FG* fluorogold, *GABA* gamma-amino-butyric-acid, *GnRH* gonadotropin release-hormone, *HRP* horseradish peroxidase, *La*t lateral hypothalamus, *LC* Locus Coeruleus, *LHN* lateral hypothalamic nucleus, *LPO* lateral preoptic nucleus, *Med* medial hypothalamus, *MPO* medial preoptic nucleus, *NA* noradrenaline, *NPY* neuropeptide-Y, *6-OHDA* 6-hydroxydopamine, *ORX* orexin, *PeVN* periventricular nucleus, *PHAL* phaseolus vulgaris leukoagglutinin, *PN* posterior nucleus, *Post* posterior hypothalamus, *PVN* paraventricular nucleus, *SCN* suprachiasmatic nucleus, *SON* supraoptic nucleus, *TB* true blue, *TMN* tuberomammillary nucleus, *VMN* ventromedial nucleus, *VPA* vasopressin, *WGA* wheat germ agglutinin

**Table 2:** Effects of DSP-4 on noradrenaline levels in different brain areas in rats

	Amygdala	Hippocampus	Hypothalamus	Striatum
Controls	6.82 ± 1.30	3.31 ± 0.68	12.84 ± 2.31	0.52 ± 0.13
DSP-4	2.23 ± 0.76*	1.21 ± 0.49*	6.98 ± 0.90*	0.43 ± 0.12

NA levels were measured in adult male Sprague Dawley rats 7 days after administration of either saline or DSP-4 (50 mg/kg i.p.) to produce a selective lesioning of NE terminals arising from the LC. NA has been assessed in homogenates of freshly dissected brain regions: the samples were prepared and assayed by HPC system coupled with coulometric electrochemical detector as previously described (Giorgi et al. 2003; Fornai et al. 1996). Results were obtained from five animals per group and are expressed as mean ± SD values, in ng/mg of protein. Differences among groups have been used using Student’s *t* test

*p < 0.01 compared with controls

**Fig. 2:**
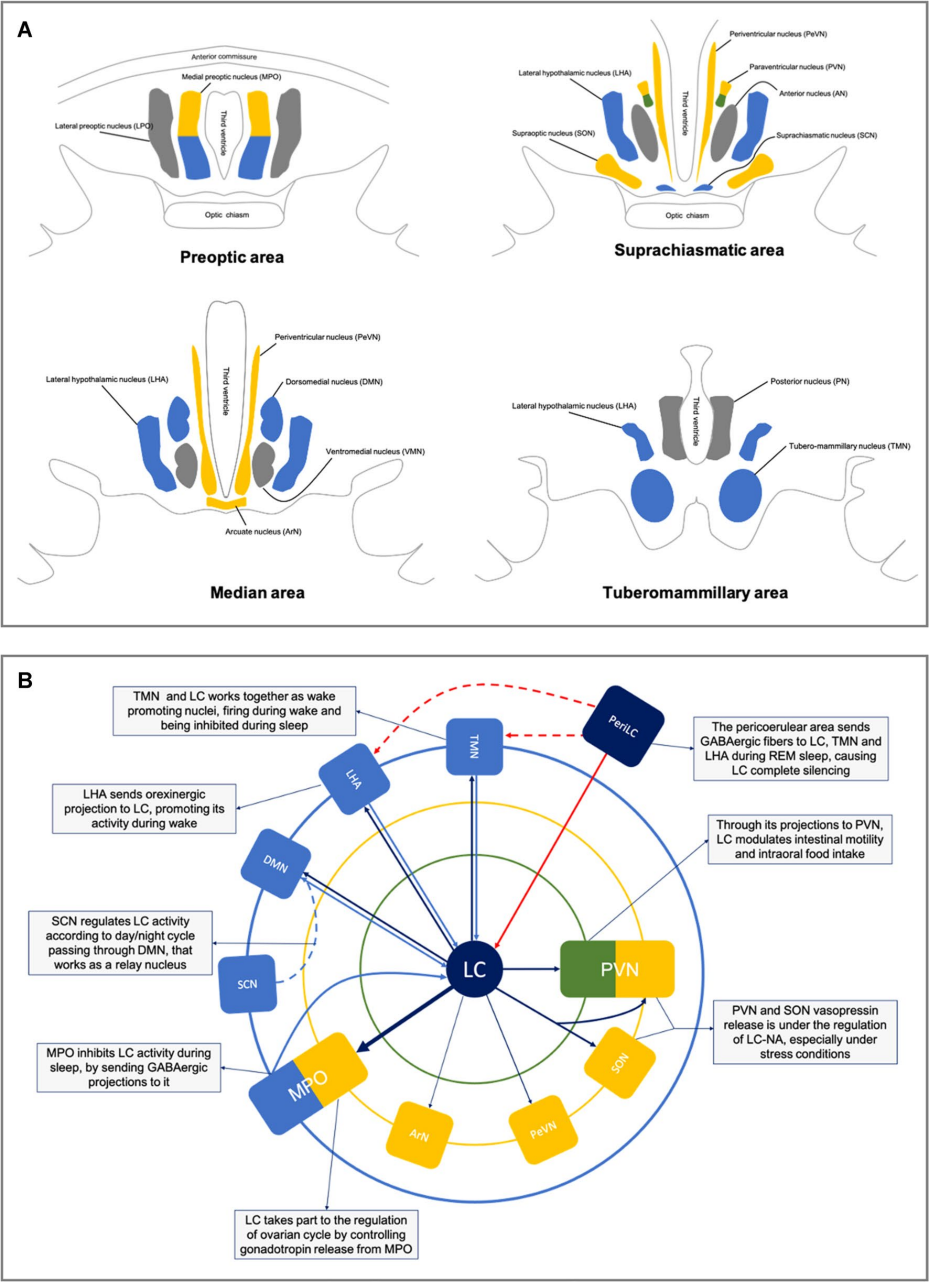
Schematic anatomy of the hypothalamus and its functional connections with Locus Coeruleus. In panel **a**, the hypothalamic nuclei are represented along the anteroposterior axis, divided into four regions: preoptic, suprachiasmatic, median and tuberomammillary area. Nuclei are colored on the basis of their main function considered in the present paper: yellow for endocrine nuclei, green for autonomic ones, light blue for sleep/wake cycle regulators. In panel **b** are represented schematically the functional connections between the Locus Coeruleus (LC) and hypothalamus. At the center of the diagram there is the Locus Coeruleus, surrounded by three concentric circles; the inner one (green line) represents the autonomic part of paraventricular nucleus, the second one (yellow line) endocrine nuclei, while the outer one (blue line) represents the sleep/wake cycle regulator nuclei. The arrows exemplify the functional connections existing between LC and each hypothalamic nuclei; moreover, also the pericoerulear zone (PeriLC) is reported, to better represent the REM sleep network. *DMN* dorsomedial nucleus, *MPO* medial preoptic area, *LC* Locus Coeruleus, *LC-NA* Locus Coeruleus noradrenergic system, *LHA* lateral hypothalamic area, *LPO* lateral preoptic area, *PeriLC* pericoerulear zone, *PVN* paraventricular nucleus, *SCN* suprachiasmatic nucleus, *SON* supraoptic nucleus, *TMN* tuberomammillary nucleus

In this section, we summarize the current knowledge on the anatomical connections between hypothalamus and LC and report the main functional effects of LC on hypothalamic nuclei, based on the available scientific literature in which the effects of LC had been estimated after its experimental lesion (Table 1). These data have been obtained by extensively reviewing published papers on this topic; in particular, we performed a PubMed search, using as keywords “hypothalamus” and “locus coeruleus”, without temporal limits. We obtained a list of 1446 studies, from which we selected only papers written in the English language and which they were specifically assessed the anatomical connections of LC with the hypothalamus (total studies selected: 80). In Fig. 2, a schematic representation of hypothalamic nuclei (A) and of the functional connections between the LC and those nuclei (B) are provided.

In detail, in the following sub-sections, we will describe for each one of the different hypothalamic sub-regions which are affected in AD, the evidences for such an involvement in patients, their anatomical/functional connections with LC, and how the LC-NA system impairment may concur to those phenomena.

### Endocrine hypothalamus: involvement in AD and potential role of LC

The occurrence of alterations of the hypothalamus-hypophysis axis has been confirmed in several studies on AD patients (Csernansky et al. 2006; Yong-Hong et al. 2013; Vest and Pike 2013). These subjects often show higher glucocorticoids plasma levels, when compared to healthy controls, in parallel with a reduced responsiveness to these hormones (Csernansky et al. 2006); moreover, corticotropin releasing-hormones (CRH) levels are altered in the cerebrospinal fluid of AD patients (Banki et al. 1992) and similar findings have been obtained concerning thyroid hormones. It has been suggested that in AD, tireotropin releasing-hormone (TRH) signaling system may not work properly, either due to a reduced release of TRH itself or to a decreased sensitivity to thyroid hormones central feedback on hypothalamus-hypophysis (Yong-Hong et al. 2013). This may cause a latent or frank hypothyroidism, which has been shown to occur more frequently in AD subjects than in age-matched cognitively intact subjects, and which by itself may also exacerbate and worsen the progression of AD (Tan and Vasan 2009; Accorroni et al. 2017).

Furthermore, endocrine impairment in AD significantly involves also the sexual hormones axis. Reduced levels of sexual hormones have been observed in AD patients and also in MCI subjects (Vest and Pike 2013); even more interestingly, in subjects at higher risk of developing AD, lower plasmatic concentrations of estrogens, in women (Manly et al. 2000) and of testosterone, in men (Moffat et al. 2004), were observed when compared to subjects at lower risk. Decreased levels of GnRH have been found in AD patients (Vest and Pike 2013) and in the preclinical animal model such a reduction was associated with cognitive impairment (Bowen et al. 2015). Interestingly, in 2013, Zhang and colleagues showed that in the brain of aged mice, the dysregulation of neuroinflammatory pathways leads to the reduction of GnRH production, which parallels cognitive impairment; noteworthy, the exogenous administration of GnRH partially restored such cognitive alterations (Zhang et al. 2013).

LC sends efferences to all of the hypothalamic nuclei which play a role in endocrine functions and it receives reciprocal afferents from these very same nuclei. LC is connected to the magnocellular system of PVN and SON and thus it may modulate vasopressin (VPA) and oxytocin (OXY) release: for their functional implications see details reported in “Involvement in AD of autonomic functions and potential role of LC” section.

More important for their implications on the endocrine system are the strong reciprocal connections of LC with the hypothalamic parvocellular system. In fact, it projects GnRH-producing cells in the MPO (Campbell and Herbison 2007), from which it receives afferent fibers (Swanson 1976; Uschakov et al. 2007). Moreover, LC sends efferents also to the periventricular nucleus (Roizen et al. 1976; Javitch et al. 1985), where parvocellular CRH-producing neurons are placed, and to the arcuate nucleus (ArN), which hosts TRH-producing neurons (Luppi et al. 1995; Yoon et al. 2013). However, it has not been shown any significant decrease of TRH release after LC lesion (Jaffer et al. 1990), and there are contrasting data concerning its effects on CRH release (Szafarczyk et al. 1985). In 1985, Szafarczyk and colleagues showed that the lesion of the ventral noradrenergic bundle causes the disappearance of the adrenocorticotropic hormone (ACTH) circadian pattern and a reduction of stress-related ACTH release; however, it is worth mentioning that they did not directly lesion the LC and these authors themselves acknowledged that such an effect could be due to the lesion of NA projections originating from other NA nuclei as well (e.g. A1 or A2) (Szafarczyk et al. 1985). Concerning growth hormone (GH), Blue-Pajot and colleagues showed an increase in GH serum levels after LC lesion (Bluet‐Pajot et al. 1992), but to our knowledge this observation was not replicated by other studies.

Conversely, several pieces of evidence suggest that LC may directly participate to gonadotropin secretion and thus its impairment might significantly affect the ovarian cycle. Endroczi and colleagues found that follicle-stimulating hormone plasma levels were reduced after LC lesion (Endroczi et al. 1978); furthermore, LC lesion affects luteinizing hormone secretion both in basal conditions and during ovulation (Dotti and Taleisnik 1982, 1984; Franci and Antunes-Rodrigues 1985; Anselmo-Franci et al. 1997; Martins-Afférri et al. 2003).

Similarly, in 2003, Martins-Afferi and colleagues observed that in rats in which LC had been selectively lesioned, increased luteinizing hormone-releasing hormone levels could be observed within the MPO and median eminence (Martins-Afférri et al. 2003); as hypothesized in the case of the magnocellular system (Rodovalho et al. 2006), the authors suggested that LC-NA may influence the release of releasing-hormones by promoting the depolarization of hypothalamic cells (Martins-Afférri et al. 2003).

In conclusion, there are strong evidences for a role of LC in the proper functioning of the gonadotropin axis. Accordingly, the loss of facilitating mechanism by LC may explain, at least in part, in AD the alteration of gonadotropin secretion by the MPO neurons surviving to neurodegenerative phenomena; this might be further amplified by the fact that LC loss might play an earlier, and even more important role, in concurring to the marked degeneration itself of MPO which occurs in AD (see “The loss of neuroprotective effects of LC efferences may enhance AD pathology also in the hypothalamus” section). Similarly, even though the available evidences concerning the direct functional effects of LC activity upon in TRH (Jaffer et al. 1990) and CRH (Szafarczyk et al. 1985) release are still scarce and contradictory, in this case LC degeneration might be crucial for the occurrence of degeneration neurons of these areas (periventricular nucleus) (see “The loss of neuroprotective effects of LC efferences may enhance AD pathology also in the hypothalamus” section).

### Body weight loss and appetite dysregulation in AD

Endocrine alterations are not the only manifestations of hypothalamic involvement in AD, as patients often experience disturbances of body weight and metabolism. Body weight loss and metabolic impairment are experienced quite commonly in patients affected by this disorder (Ishii and Iadecola 2015). Hyporexia and weight loss may precede AD clinical onset, and some studies even associated the risk of developing AD with a lower body mass index (Jimenez et al. 2017). Even though the causes of these alterations are not fully understood, it is widely accepted that hypothalamic structural/functional alterations occurring in AD might be involved (Ishii et al. 2014); in particular, the disruption of the connections between the hypothalamus and specific hormones, such as leptin should be taken in consideration (Marwarha and Ghribi 2012). Leptin is an adipocyte-derived hormone, whose main known role is to imbalance energy demand and body weight, reducing appetite acting on neuropeptide-Y-containing neurons of ArN (Espinoza García et al. 2021). Both clinical and pre-clinical pieces of evidence support a potential role of the impairment of leptin-signaling system in the genesis of body weight loss in AD. In patients, abnormally low levels of plasmatic leptin were observed (Holden et al. 2009), and in an AD animal model a reduced sensitivity of hypothalamus to leptin itself was found (Ishii et al. 2014); the latter phenomenon may result in the dysregulation of appetite, in particular in low calories intake despite the reduction of body weight (Ishii and Iadecola 2015).

Through its connection with PVN and ArN, LC may take also part in appetite and intraoral food intake regulation. In 2001, Ammar and colleagues administered rats with DSP-4, to selectively lesion the LC, and then they closely monitored their feeding behavior. They found out that LC-lesioned animals were less prone to look for food and had a lower daily caloric intake; moreover, they also found that intracerebroventricular NA administration reinstated normal appetite in previously LC-lesioned rats (Ammar et al. 2001). Thus, LC-NA projections to PVN may be crucial for promoting appetite and calories assumption (Ammar et al. 2001). As said, weight loss and body mass reduction can occur early in the natural history of AD, and may even precede the onset of neurological manifestations (Ishii and Iadecola 2015; Jeong et al. 2020); it may be thus conceivable that such metabolic impairment may be one of the first signs of LC-hypothalamus pathology, also considering the early involvement of LC in AD (Braak et al. 2011; Kelly et al. 2017).

### Involvement in AD of autonomic functions and potential role of LC

In AD there are several evidences for a higher incidence blood pressure (BP) abnormalities and gastrointestinal dysfunctions (Idiaquez and Roman 2011; Hughes et al. 2018). In particular, among BP alterations, those most frequently reported in AD patients concern orthostatic hypotension and alterations in circadian BP variability (Chen et al. 2013; Isik et al. 2019). AD patients were observed to show orthostatic hypotension with a rate similar to dementia with Lewy Bodies (DLB) patients, even though the former ones do not often complain symptoms related to low BP (Isik et al. 2019). Moreover, it was observed that AD patients have higher mean BP, during both wake and sleep (Chen et al. 2013); this may result from both an alteration of BP modulation, mediated also by the hypothalamic PVN, and the dysregulation of circadian BP variation. Nevertheless, surprisingly only a very few studies had evaluated the role of hypothalamus in the genesis of autonomic dysfunction in AD; among them, are worth mentioning the findings by Burke and colleagues, which in 1994, performed a post-mortem study to evaluate the involvement of specific hypothalamic regions in AD patients which had experienced BP abnormalities and hypotension during their life (Burke et al. 1994). Intriguingly, they found the occurrence of massive accumulations of NFT in the PVN, together with a reduction of phenylethanolamine *N*-methyltransferase activity within hypothalamic neurons (Burke et al. 1994).

Concerning the gastrointestinal alterations that occur in AD, these mainly consist of gastric constipation, impaired intestinal motility and stypsis (Idiaquez and Roman 2011). These alterations have been receiving growing attention in recent years, for the possible link between enteric nervous system and NDDs (Pellegrini et al. 2018). In particular, it has been suggested that the disruption of the enteric epithelial barrier and consequent local inflammation may impair gastrointestinal motility and, at the same time, promote the diffusion of the inflammatory processes to the central nervous system, thus triggering neurodegeneration (Pellegrini et al. 2020). On the other hand, from a neurophysiological point of view, intestinal motility is strictly related with the proper functioning of the dorsal motor nucleus of vagus. However, also the hypothalamus, specifically the PVN, has been shown to play a relevant role in gastrointestinal motility (Bonaz et al. 1992a, b) and, as already mentioned above PVN is affected in AD (Mann et al. 1985; Swaab et al. 1992; Baloyannis et al. 2015).

Both the hypothalamus and the LC play a crucial role in modulating the activity of the autonomic nervous system. LC is likely to exert most of these regulating functions through the direct connections it has with other nuclei of the reticular formation (Fornai and Ferrucci 2017). However, LC and PVN, which is considered as the core of the autonomic hypothalamus, are also densely interconnected to one another (Cedarbaum and Aghajanian 1978; Baldo et al. 2003) (Table 1) and some lesioning studies performed in the last decades have contributed to better clarify their functional link.

Concerning the interaction of LC with hypothalamic control of gastrointestinal function, in 1992 Bonaz and colleagues showed in rats that LC lesion causes a reduction in intestinal motility (Bonaz et al. 1992b): since such a function is known to be strongly modulated by PVN as well, through its connection to the dorsal motor nucleus of the vagus (Flanagan et al. 1992), a contribution of this indirect connection to the effect of LC lesion could be hypothesized independently from the concomitant role of the direct projection of LC to dorsal motor nucleus of vagus (Samuels and Szabadi 2008). To further test this hypothesis, Bonaz and colleagues performed another study in which they lesioned NA terminals in the PVN by locally injecting 6-hydroxydopamine; by this approach, they showed a reduction of intestinal motility similar to the one found after whole LC lesion (Bonaz et al. 1992a), thus ruling out any significant contribution to this phenomenon of the direct connection of LC with extra-hypothalamic regions modulating the autonomic nervous system. Thus, through these mechanisms LC lesion may be involved in the pathogenesis of gastrointestinal dysfunctions occurring in AD, such as constipation and gastric retention.

Finally, the strict relationship occurring between LC and magnocellular neurons of PVN might be relevant for the BP alterations experienced by subjects with AD. In particular, axons originating from LC neurons densely innervate the PVN, and they specifically target its VPA-ergic neurons (Sawchenko et al. 1985; Reyes et al. 2005); at the same time, LC receives VPA-ergic afferent fibers from PVN (Hawthornet al. 1985). Accordingly, it has been shown that the experimental lesion of LC causes alterations in VPA and OXY secretion. From a functional point of view, it has been shown by Shih and colleagues that projections from neurons belonging to PVN to LC modulate the baroreceptor stimulation reflex (Shih et al. 1995). In fact, the lesion of PVN reduces the strong inhibitory effect that LC stimulation exerts on the baroreceptor reflex (Shih et al. 1995). Interestingly, in 1984, Banks and colleagues had shown that LC lesion by the focal microinfusion of 6-hydroxydopamine abolishes the physiological reduction of SON discharge frequency, which usually follows the stimulation of carotid sinus baroceptor (Banks and Harris 1984), and SON is another magnocellular hypothalamic nucleus which receives dense innervation by the LC.

Again, in line with the role of connection between LC and magnocellular hypothalamic nuclei, in 2006 Rodovalho et al. showed a reduction in VPA secretion after LC lesion in a rat model of cerebral hemorrhage (Rodovalho et al. 2006). Thus, it is likely that LC modulates systemic BP through a dense and complex neuronal network, in which PVN represents an important node, both considering the magnocellular neuroendocrine compartment and the autonomic one (Banks and Harris 1984; Samuels and Szabadi 2008).

For the sake of completeness, it is worth mentioning in this context that autonomic symptoms occurring in AD have been also explained in light of the degenerative phenomena involving the autonomic centers of brainstem and spinal cord (Idiaquez and Roman 2011; Coon et al. 2018), and that LC is strongly connected also with these structures (Samuels and Szabadi 2008), and this might represent another key pathway through which the LC degeneration might concur to dysautonomic disturbances occurring in AD patients: nevertheless, a detailed description of these connections and of their alterations in AD is beyond the aim of the present review. By the same token, LC was found to modulate intestinal motility also through its direct projections to the dorsal motor nucleus of vagus (Samuels and Szabadi 2008), which may concur, together with its indirect effect through PVN (Bonaz et al. 1992a, b) to the mechanism of LC modulation of enteric nervous system.

### Circadian rhythm and sleep/wake cycle: potential role of LC-hypothalamic interactions

LC is considered crucial in the regulation of sleep/wake cycle and in the modulation of the circadian rhythm. LC takes part to a complex network involved in sleep homeostasis which includes a number of hypothalamic nuclei, namely SCN, MPO, DMN, VMN, the Lateral Hypothalamic Area (LHA) and TMN (for a review see Berridge et al. 2012) (Fig. 2). In brief, the retinal-hypothalamic tract, which conveys information on environmental light conditions, reaches the SCN. Neurons belonging to the latter nucleus target GABAergic neurons in the MPO, thus modulating their activity according to circadian rhythm. These neurons in turn project to the so-called “wake promoting nuclei”, which include indeed the LC, together with the pedunculopontine nucleus, the dorsal and the TMN, inhibiting their activity and thus promoting sleep.

AD patients suffer from significant disturbances of the sleep-waking cycle. These include, but are not limited to, inversion of the sleep/wake cycle and sleep structure breakdown and fragmentation (Wu et al. 2019). In particular, in AD patients it has been observed a reduction of the rapid eye movements (REM) and of the slow-wave sleep stages (Prinz et al. 1982); this results in a non-restoring night sleep, which further impairs cognitive performances and causes daytime sleepiness (Wu et al. 2019). Moreover, the whole circadian rhythm is altered in AD; clinical studies demonstrated that AD patients show abnormal thermoregulation along daytime and disrupted circadian rest-activity rhythm (Harper et al. 2001; Saper 2013).

The degeneration of specific hypothalamic nuclei occurring in AD is likely to be key in the dysregulation of circadian rhythms and sleep disorders (Ishii and Iadecola 2015; Musiek et al. 2015); this is particularly true in the case of the SCN (Harper et al. 2008), but also considering other hypothalamic nuclei involved in the sleep cycle, such as the TMN, the DMN and the VMN (Hiller and Ishii 2018).

LC establishes reciprocal connections with each one of these hypothalamic nuclei (i.e. the SCN, MPO, DMN, VMN, the LHA and the TMN). The SCN sends efferents to the LC (Hawthorn et al. 1985; Krout et al. 2002), both directly and through its connection with DMN and VMN, which are also connected with the LC (Cedarbaum and Aghajanian 1978; Baldo et al. 2003). As already said above, GABAergic neurons belonging to the MPO sends efferent fibers to the LC (Swanson 1976; Uschakov et al. 2007), and through this pathway MPO might contribute to sleep induction. On the other hand, LC projects to the wake-promoting TMN (Farley and Hornykiewicz 1977; Sobrinho and Canteras 2011).

During wakefulness, LC neurons can discharge either tonically or phasically, while during NREM sleep LC discharge is dramatically reduced, and is completely suppressed during REM sleep (Berridge et al. 2012). REM sleep is characterized by cortical EEG desynchronization, muscle atonia and rapid eye movements; such a state is mediated by a neural network controlled by the so-called “REM sleep center”, which in humans has been identified with the peri-coerulear/sub-coerulear region and is strictly synaptically and functionally connected with LC (Peever and Fuller 2017). Glutamatergic/glycinergic neurons placed within the REM sleep center send their projections to motor nuclei in the spinal cord, while efferent fibers from its GABAergic neurons reach wake-promoting nuclei such as LC, TMN and dorsal raphe nucleus; in this way, the REM sleep center induces muscle atonia and maintains sleep state, despite the high desynchronization of cortical activity due to concomitant basal forebrain cholinergic nuclei activation (Peever and Fuller 2017). REM sleep center inhibits also the LHA, mainly by targeting its orexinergic neurons, which can also be found in the DMN and in the TMN (Peever and Fuller 2017). Orexin (ORX) is a key neurotransmitter of the wake state, and orexinergic neurons of hypothalamus project to LC, thus promoting the transition from sleep to wake and wakefulness maintenance (Peever and Fuller 2017). LC and LHA are densely interconnected (Peyron et al. 1998; Horvath et al. 1999) and the integrity of such a network is crucial for sleep/wake cycle proper functioning and sleep phases alternation (Tortorella et al. 2013). A specific degeneration of ORX-containing neurons causes narcolepsy (Mahoney et al. 2019), which is a disease featured by excessive diurnal somnolence and sudden muscle atonia. However, despite the huge amount of data on the role of both LC and hypothalamus in sleep regulation and circadian rhythm modulation, only a few lesion studies are available in current literature, exploring specifically the reciprocal interplay between the two structures. Among these, in 2004, Blanco-Centurio and colleagues lesioned the LC of rats using anti-DBH antibodies linked to the neurotoxic enzyme saporin and they observed, as an effect, an increased sleep during the dark period and increased limb movements during REM sleep (Blanco-Centurion et al. 2004). They also showed that when they selectively lesioned those LC neurons which are targeted by ORX fibers (by the microinfusion of saporin-linked anti-ORX receptor antibodies), such an alteration of sleep pattern was even more evident, despite a more restricted LC-NA neurons damage (Blanco-Centurion et al. 2004). The authors explained these results in light of the ORX projections to LC and peri-LC area. In line with these findings, in 2016 Schwartz and colleagues showed that in rats the lesion of LC reduces the efficacy of almorexant, an ORX receptor antagonist which is used in narcolepsy to promote REM sleep and thus to regulate sleep/wake cycle; they interpreted their findings as a proof of the importance of LC-ORX system in REM sleep and sleep cycle (Schwartz et al. 2016).

While no study, to our knowledge, was designed specifically to assess post-mortem, in AD patients, the potential co-operation of LC degeneration with specific hypothalamic alterations concerning the vegetative, endocrine and metabolic function described in above sections, this has been done post-mortem concerning correlation of LC with sleep-related hypothalamic degeneration. In particular, to specifically assess the role of the alteration of LC-hypothalamic connections in the pathogenesis of AD sleep disturbances, recently Oh and colleagues, performed a stereological analysis of AD brains, evaluating the pathological involvement of wake-promoting nuclei, and particularly focusing on LC, TMN and LHA (Oh et al. 2019). They found a marked degeneration of all these three nuclei; in particular, the authors observed a dramatic reduction of the NA neuronal population in the LC, of ORX-producing neurons in the LHA, and of histaminergic cells in the TMN. Neuronal death was associated with increased pTau accumulation at the level of all of the three nuclei, suggesting the occurrence of a common pathogenetic pathway, likely related to NFT pathology (Oh et al. 2019).

Another very interesting study on the potential role of LC degeneration in the genesis of sleep alterations in AD was performed post-mortem in humans by Kasanuki and colleagues in 2014. These authors assessed the neuronal ORX population of LHA in parallel with the NA neurons of LC in the brain of subjects affected by AD or by DLB, and correlated their findings with amyloid, tau and synuclein pathology (Kasanuki et al. 2014). They found a marked reduction of LHA-orexinergic neurons in both types of dementia, which was associated with a quantitatively comparable neuronal loss in LC; moreover, they observed that ORX projections to LC were dramatically reduced in AD and DLB, as well as NA fibers targeting LHA (Kasanuki et al. 2014). Interestingly, the authors did not find any relation between the degree of LHA neuronal loss and amyloid or synuclein pathology (which is considered more typical of DLB pathology than of AD), while they showed a strong correlation with tau pathology burden. Remarkably, while they showed the occurrence of NFT within ORX neurons, they could not find any amyloid plaques nor Lewy bodies (which are classically considered the hallmark of “synucleinopathies” including DLB) within orexinergic nuclei (Kasanuki et al. 2014). These findings further suggest that the hypothalamic damage occurring in AD may be associated with tau pathology, which may start indeed in the LC itself (Braak and Del Tredici 2011) (see “The occurrence of neuronal loss/degenerative phenomena and NE alterations in the hypothalamus of AD patients” section).

All of these factors may contribute to the disruption of LC-hypothalamic network which regulates sleep/wake cycle, and they may account for the several sleep disorders that occur in NDDs, such as RBD in parkinsonisms (St Louis and Boeve 2017) and circadian alteration in AD (Musiek et al. 2015).

Finally, it is worth mentioning in this context also a recent exciting theory which has been proposed to explain a further pathogenetic link between LC and sleep disorders, i.e. that the disruption of the sleep cycle itself may further concur with the ongoing LC and hypothalamus degeneration mechanisms. In particular, based on experimental data it has been proposed that the fragmentation of night sleep enhances NFT-related AD pathology both in LC neurons and ORX neurons of LHA. In 2016, Zhu and colleagues submitted wild-type mice to intermittent short sleep, and showed the occurrence of cell loss within the LC and the LHA and the concomitant reduction of their fronto-cortical projections (Zhu et al. 2016). The same group strengthened these observations in a tau-pathology mouse model, in which they found a dramatical worsening of tau-pathology in the LC, associated with marked neuronal loss, after prolonged sleep fragmentation. In these mice, the burden of NFT, neuroinflammation and neuronal death was increased also in forebrain regions, especially in limbic cortex (Zhu et al. 2018). Thus, it may be hypothesized that LC-hypothalamic impairment may establish a vicious cycle, in which the negative effects on sleep hygiene further hamper LC integrity, which in turn enhances detrimental consequences on the hypothalamus.

## Discussion and conclusions

In this review, we aimed to analyze the current evidences for a role of LC in hypothalamic alterations occurring in AD. This is based on the consolidated data concerning both a marked LC impairment and a significant hypothalamic alteration, as well as on the known strong reciprocal anatomical connections linking these two brain structures. It is worth remarking, once again, that LC has been clearly demonstrated to be already degenerated in AD patients years before the onset of degenerative phenomena in other parts of the brain, and of cognitive symptoms. Thus, LC degeneration might be key in concurring to the onset of a variety of neurological and extra-neurological symptoms, as well as in concurring to the degenerative phenomena taking place in other brain areas in AD. However, the extensive analysis of the above-discussed literature shows that, while the impairment of LC and hypothalamus in AD have been studied quite in detail separately from one another, thus far the specific involvement of their reciprocal connection in AD pathogenesis has not been specifically assessed.

We described the available evidences for an effect of LC degeneration on specific hypothalamic functional alterations, hypothesizing that the pathological degeneration of LC in AD might be “simulated” by selectively lesioning experimentally the LC in animal models. Among the functions which are considered to be controlled by the hypothalamus, we provided clear evidences that LC-NA degeneration is sufficient by itself to induce: (a) gonadotropin release reduction; (b) hyporexia and reduced food intake; (c) BP regulation impairment; (d) alterations in gastrointestinal motility; (e) significant alterations of the sleep–wake cycle. Remarkably, all of these alterations have been reported to occur more frequently in AD patients than in age-matched cognitively intact persons. Even more, there are clear evidences for a specific role of the loss of those LC-NA fibers specifically projecting to hypothalamic nuclei, at least concerning GnRH alterations (i.e. the MPO), gastrointestinal dysfunction (the PVN), baroreceptor reflex (i.e. PVN and SON). Concerning other hypothalamic nuclei, there are strong hints for such a functional correlation between LC degeneration and an impairment of their proper functioning.

Thus, we think that the loss of LC-NA fibers terminals in the hypothalamus, which constantly occur in AD, is sufficient, by itself, to induce the functional alteration of specific hypothalamic nuclei, even in the case that the latter ones are not directly involved by AD pathology.

However, we also hypothesize that in AD there is a significant chance that, not only LC degeneration might be key in altering the function of specific hypothalamic nuclei, but it might indeed bear a causative role on the degeneration of such nuclei, as there is a huge amount of literature showing a neuroprotective role of LC-NA toward neurodegeneration/neurotoxicity in its target areas. To support this hypothesis, we reported the available post-mortem evidences for a marked cell loss in specific hypothalamic nuclei in AD, namely the SCN, the PVN, the DMN and VMN, and the TMN (Mann et al. 1985; Swaab et al. 1992; Thal et al. 2002; Braak et al. 2011; Baloyannis et al. 2015; Hiller and Ishii 2018; Oh et al. 2019). Thus, we hypothesize that whatever may be the precise mechanisms through which LC modulates the degeneration of its target hypothalamic regions, at least for some of these there might be a direct causative role of LC terminal loss on neuronal degeneration. Remarkably, very recent post-mortem analysis in patients with dementia have directly put in relation the degeneration of LC with that of nuclei involved in sleep regulations such as TMN (Oh et al. 2019) and LHA (Kasanuki et al. 2014; Oh et al. 2019).

Unfortunately, to our knowledge thus far there have not been systematic studies in experimental AD models in which the effects of selective LC lesion on the degeneration of specific hypothalamic nuclei have been assessed. These are experiments which we think are urgently needed for verifying the existence of such a pathogenic link. By this systematic approach, it might also be shown that hypothalamic neurons, which are altered in AD, but whose physiological functions are not modified by the sole LC experimental lesion (e.g. TRH and CRH producing neurons, see “Endocrine hypothalamus: involvement in AD and potential role of LC” section) might actually be impaired in AD due to a proneness to degenerate in the absence of LC-NA. Thus, the clinical consequences of their degeneration might be related only to a structural alteration and not to a “functional” one related to LC impairment.

Thus far, a potential mechanism through which LC has been directly put in causative pathogenic relation with the degeneration of hypothalamic nuclei has been the prion-like spreading of Tau-related pathology from LC neurons up to its target areas, which include the hypothalamus (Braak and del Tredici 2011; Kasanuki et al. 2014; Iba et al. 2015). Another well-known mechanism by which LC loss induces neurodegeneration is neuroinflammation: the fact that both GnRH releasing neurons and ORX neurons are very sensitive to neuroinflammation causing their impairment (Grossberg et al. 2011; Zhang et al. 2013), might indeed indirectly reveal a causative link between LC degeneration and the degeneration in these nuclei in AD. To our knowledge, the other mechanisms through which LC degeneration might induce/potentiate selective degeneration of specific hypothalamic nuclei have not been studied in detail and systematically so far. This is another set of experimental studies which, in our opinion would be mandatory to better clarify the pathogenetic link between LC loss and hypothalamic degeneration in AD models.

Stepping back to the two mechanisms which have been confirmed so far as involved in LC loss-hypothalamic degeneration pathogenetic link, i.e. prion-like p-Tau spreading and neuroinflammation potentiation, these bear already potential interesting therapeutic implications. In fact, in this scenario, the degeneration of hypothalamic nuclei might start very early in the course of the AD, when LC neurons are not yet markedly degenerated, but they already bear a significant burden of pathological proteins (such as for instance phospho-Tau); despite the recent failure of phase II and III controlled trials aimed at slowing amyloid accumulation early in the course of AD, there are currently new studies assessing the role of anti-Tau protein monoclonal antibodies in AD (Congdon and Sigurdsson 2018; Dehay et al. 2015), which might potentially help to slow down also the degeneration in LC target areas, including the hypothalamus. Similarly, concerning neuroinflammation, early anti-inflammatory treatment which are currently under study in AD (Ozben and Ozben 2019) might be potentially useful also in slowing down hypothalamic alterations associated with this disorder; indeed, NA-related drugs themselves might be even proposed as a powerful anti-inflammatory treatment (Kalinin et al. 2006).

Finally, a better understanding, in experimental models and in AD patients, of the relationship existing between LC and hypothalamus pathology may potentially lead also to identifying new diagnostic early disease biomarkers. In fact, many of the dysfunctions that may be related to LC-hypothalamic pathology occur early in AD patients’ clinical history (Ishii and Iadecola 2015) and could be detected in the prodromal- or even asymptomatic-stages of the disease. An interesting opportunity is represented by the involvement of the hypothalamic-sexual hormones axis; as abovementioned, the influence of LC on GnRH has been clearly showed in pre-clinical studies, and GnRH levels alterations appear early in AD progression, in parallel with LC degeneration. Thus, sexual hormones alterations and gynecological disorders may deserve particular attention in studies on middle-aged/elderly persons, to clarify whether they may represent or not good candidates as prodromal symptoms for AD, being this particularly crucial in the context of a gender-tailored medicine.

Interestingly, promising LC neuroimaging biomarkers have also been developed recently, which for the first time may allow to evaluate human LC-hypothalamus pathology in vivo. Indeed, LC can be studied by MRI profiting of T1-weighted neuromelanin-sensitive sequences (Galgani et al. 2020; Liu et al. 2017), and this approach has already been applied to assess LC involvement in healthy elderly subjects (Liu et al. 2020), as well as in patients affected by several neurological disorders, including AD, PD, REM-behavior disorder, Multiple system Atrophy, chronic traumatic encephalopathy essential tremor (e.g., García-Lorenzo et al. 2013; Matsuura et al. 2013; Isaias et al. 2016; Betts et al. 2019). Positron Emission Tomography tracers specific for NA terminal transporters are also under development, such as ^11^C-MeNER, which has been tested already in PD patients (Sommerauer et al. 2018; Doppler et al. 2021). Those imaging techniques may be used in the near future to directly evaluate LC-NA system integrity in specific target regions, including the hypothalamus (Brumberg et al. 2019; Andersen et al. 2020). Thus, it might be that in the near future the combined examination of LC imaging biomarkers by MRI and Positron Emission Tomography, together with an analysis of hypothalamic biomarkers (e.g. sexual hormones alteration), might allow an earlier diagnosis in cognitively intact subjects affected by pre-symptomatic AD.

## Data Availability

Upon request.
